# Along- and Cross-Track Relocation for Ground Moving Target in a Squint Multichannel SAR System

**DOI:** 10.3390/s26113372

**Published:** 2026-05-26

**Authors:** Zuzhen Huang, Aifang Liu, Rui Zhang, Long Li, Jinjian Cai

**Affiliations:** 1 Nanjing Research Institute of Electronic Technology, Nanjing 210039, China; hzzhit@126.com (Z.H.); ssszhangrui@163.com (R.Z.); caijinjian1993@foxmail.com (J.C.); 2 School of Electronics and Information Engineering, Harbin Institute of Technology, Harbin 150001, China; lilong19970311@163.com

**Keywords:** ground moving target indication, target relocation, squint synthetic aperture radar (SAR)

## Abstract

The squint synthetic aperture radar (SAR) offers flexible beam pointing control and a wider range of applications compared to the side-looking SAR. Unlike the latter, ground moving targets exhibit shifts in both along-track and cross-track directions in squint SAR systems. To address this issue, a two-dimensional relocation method for moving targets is proposed in this paper. Firstly, the shift characteristics of moving targets in squint SAR systems are analyzed, revealing that the two-dimensional location shifts are correlated with both the target’s radial velocity and its imaging location. The proposed algorithm initially performs clutter suppression on the SAR imagery and estimates the radial velocity of the moving target. The two-dimensional location information is then derived by solving a set of joint equations. Finally, some numerical experiments are provided to demonstrate the effectiveness of the proposed method in the squint SAR system.

## 1. Introduction

The accurate relocation of ground moving targets is a critical step in synthetic aperture radar (GMTI) processing [1,2,3,4]. While stationary targets are correctly geolocated in SAR imagery, the motion-induced phase modulations of moving targets cause their imaged positions to deviate from their true geographic locations. In traditional side-looking SAR systems, this shift is predominantly one-dimensional, manifesting as an along-track shift. Consequently, established relocation methods for side-looking SAR primarily focus on estimating and correcting this azimuthal shift [5,6,7].

In 2016, Zhang et al. introduced an instantaneous interferometry-based SAR ground moving target indication method that enhanced the accuracy of relocation estimation via iterative computation and time-domain interferometry [8]. In 2019, Li et al. developed a novel approach for motion parameter estimation and relocation in airborne three-channel circular stripmap SAR GMTI systems [9]. This method capitalizes on the along-track interferometry (ATI) phase and the quadratic-term coefficient in the range equation to deduce the motion and location parameters of the target. This method employs an iterative strategy to resolve the coupling effect among these parameters. In 2021, Huang et al. proposed an innovative along-track relocation technique using the non-coregistrated interferometric phase [10]. This technique circumvents the issue of along-track relocation ambiguity based on the correlation between the interferometric phase of the target without coregistration and its initial along-track position, obtaining a higher accuracy. Subsequently, in 2022, Huang et al. implemented the generalized amplitude and phase weighting technique, achieving precise relocation of ground-based moving targets [11].

However, these conventional algorithms are not directly applicable to squint SAR configurations. In squint mode, the pronounced coupling between range and azimuth signal histories results in two-dimensional shift—significant shifts in both along-track and cross-track directions [1,2,12]. This complexity is further heightened by the substantial Doppler shifts introduced by high squint angles. In 2016, Jing et al. introduced a forward-looking array-based rotatable cross-track interferometry SAR system for moving target relocation, and later extended it to squint-looking GMTI applications [13,14]. More recently, Li et al. proposed a road-network-information-assisted relocation method for multi-satellite formation SAR-GMTI, which compensates for height-induced positioning bias using prior geographic data [15]. In 2024, Li et al. developed a joint localization and tracking approach for BiSAR-GMTI via transmitter–receiver trajectory extraction [16], while Tang et al. proposed a back-projection-based motion parameter estimation scheme for dual-channel SAR, demonstrating improved accuracy in both side-looking and squint geometries [17].

To address this gap, this paper proposes a two-dimensional relocation method within a multichannel squint SAR framework. Unlike the road-network-assisted method [15] that requires prior geographic information, or the BiSAR-GMTI approach [16] that relies on trajectory inversion for both platforms, the proposed method operates solely on the target’s imaged coordinates and its estimated radial velocity. Furthermore, in contrast to the back-projection-based parameter estimation scheme [17], this work directly resolves the two-dimensional coupling by solving a derived set of equations, yielding an analytical solution for the positional shifts. The complete workflow includes clutter suppression, radial velocity estimation, jointly solving the equations, and true coordinate recovery, all conducted within the image domain using standard multichannel data.

The remainder of this paper is organized as follows. Section 2 details the signal model and derives the two-dimensional shift characteristics. Section 3 introduces the proposed relocation algorithm. Section 4 presents a quantitative analysis and simulation results to validate the method. Finally, Section 5 concludes the paper.

A complete list of the symbols used in this paper is provided in Table 1 below.

## 2. Signal Model and Shift Analysis of Moving Targets in Squint SAR

**Figure 1:**
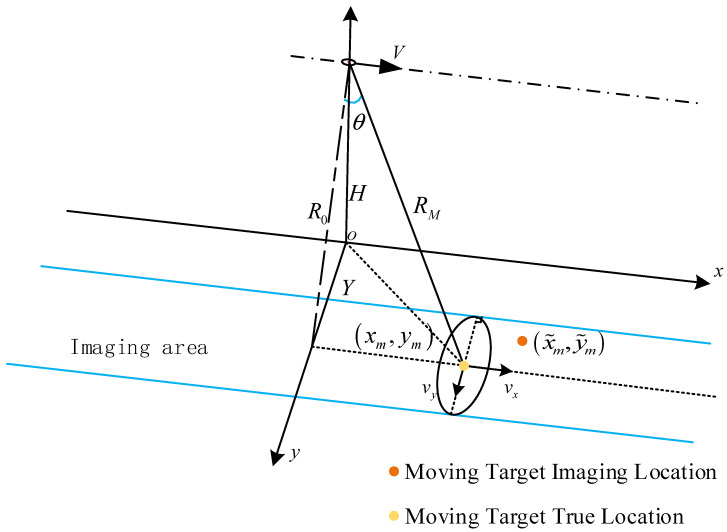
Imaging geometry of the squint SAR system.

The imaging geometry of the squint SAR system is illustrated in Figure 1, where the squint angle is denoted by *θ_sq_* (to avoid confusion, note that this is distinct from the off-nadir or incidence angle commonly represented by *θ*). The platform velocity is *V*, the flight altitude is *H*, and the shortest ground range from the radar to the scene center is *Y*. A Cartesian coordinate system is defined with the *x*-axis aligned with the along-track (azimuth) direction and the y-axis aligned with the cross-track (range) direction. A moving point target is located at an initial true position $\left(x_{m},y_{m}\right)$, with along-track and cross-track velocity components denoted by $v_{x}$ and $v_{y}$, respectively. The corresponding imaged position of the moving target is denoted by $\left({\tilde{x}}_{m},{\tilde{y}}_{m}\right)$.

Without loss of generality, the slow time *t* = 0 is defined as the instant when the antenna beam center illuminates the target. The instantaneous slant range *R*(*t*) between the radar and the moving target can therefore be expressed as:

(1) $$R(t)=\sqrt{H^{2}+{\left(y_{m}+v_{y}t\right)}^{2}+{\left(R_{0}\tan\theta_{sq}+v_{x}t-Vt\right)}^{2}},$$

where *t* represents the slow time, H denotes the radar flight altitude, and Y denotes the shortest ground range from the radar to the scene center, and $R_{0}$ represents the shortest range from the radar to the scene center that can be expressed as follows:

(2) $$R_{0}=\sqrt{H^{2}+Y^{2}}.$$

According to the geometric relationship, the target radial velocity—the component of its velocity vector along the line-of-sight—can be calculated as follows:

(3) $$v_{r}=\frac{y_{m}v_{y}+v_{x}R_{0}\tan\theta_{sq}}{R_{M}},$$

where $R_{M}$ represents the slant range when the beam center illuminates the moving target, and can be expressed as follows:

(4) $$R_{M}=\sqrt{H^{2}+y_{m}^{2}+{\left(R_{0}\tan\theta_{sq}\right)}^{2}}.$$

In SAR imaging algorithms, a target is focused at the slant range *R_M_* corresponding to the beam center moment, i.e., the nearest squint range. For side-looking SAR, *R_M_* = *R*_0_. For squint SAR, *R_M_* is the minimum value of R(t), whose square can be written as follows.

(5) $$R^{2}(t)=H^{2}+{\left(y_{m}+v_{y}t\right)}^{2}+{\left(R_{0}\tan\theta_{sq}+v_{x}t-Vt\right)}^{2}$$

Since Equation (5) is a quadratic function, its minimum and the corresponding time instant can be derived as follows:

(6) $$R_{min}^{2}\left(t_{d}\right)=R_{M}^{2}-\frac{{\left[y_{m}v_{y}+\left(v_{x}-V\right)R_{0}\tan\theta_{sq}\right]}^{2}}{{\left(v_{x}-V\right)}^{2}+v_{y}^{2}},$$

(7) $$t_{d}=-\frac{y_{m}v_{y}+v_{x}R_{0}\tan\theta_{sq}}{{\left(v_{x}-V\right)}^{2}+v_{y}^{2}}+\frac{VR_{0}\tan\theta_{sq}}{{\left(v_{x}-V\right)}^{2}+v_{y}^{2}},$$

Here, $R_{min}$ represents the slant range of the imaging location of the moving target and $t_{d}$ represents the along-track time shift. Moving target initial position location $\left(x_{m},y_{m}\right)$, with along-track and cross-track velocity components denoted as $v_{x}$ and $v_{y}$, respectively. Substituting the expression for radial velocity from Equation (3) into Equations (6) and (7) yields:

(8) $$R_{min}^{2}\left(t_{d}\right)=R_{M}^{2}-\left(\frac{v_{r}^{2}R_{M}^{2}}{{\left(v_{x}-V\right)}^{2}+v_{y}^{2}}-\frac{2v_{r}R_{M}VR_{0}\tan\theta_{sq}}{{\left(v_{x}-V\right)}^{2}+v_{y}^{2}}+\frac{{\left(VR_{0}\tan\theta_{sq}\right)}^{2}}{{\left(v_{x}-V\right)}^{2}+v_{y}^{2}}\right),$$

(9) $$t_{d}=-\frac{v_{r}R_{M}}{{\left(v_{x}-V\right)}^{2}+v_{y}^{2}}+\frac{VR_{0}\tan\theta_{sq}}{{\left(v_{x}-V\right)}^{2}+v_{y}^{2}}.$$

Considering that the ground moving target’s velocity is typically much lower than the platform velocity ($v_{x}\ll V$, $v_{y}\ll V$), Equations (8) and (9) can be simplified to:

(10) $$R_{min}^{2}\left(t_{d}\right)=R_{M}^{2}-\left[\frac{v_{r}^{2}R_{M}^{2}}{V^{2}}-\frac{2v_{r}R_{M}R_{0}\tan\theta_{sq}}{V}+{\left(R_{0}\tan\theta_{sq}\right)}^{2}\right],$$

(11) $$t_{d}\approx -\frac{v_{r}R_{M}}{V^{2}}+\frac{R_{0}\tan\theta_{sq}}{V}.$$

It is worth noting that the above expression can be decomposed into two parts: one is the position shift $-\frac{v_{r}R_{M}}{V^{2}}$ caused by target motion, and the other is the position shift $\frac{R_{0}\tan\theta_{sq}}{V}$ caused by the radar squint. Since the shift induced by the squint is identical for both the scene and the target, it produces no relative shift and therefore does not affect the relocation process. From Equation (11), the along-track shift of the moving target can be derived as:

(12) $$\Delta x_{m}=Vt_{d}-R_{0}\tan\theta_{sq}=-\frac{v_{r}R_{M}}{V}.$$

For the side-looking case, $R_{M}=R_{0}$, Equation (12) reduces to $\Delta x_{m}=-\frac{v_{r}R_{0}}{V}$, which is consistent with the description in [4,10]. Moreover, from Equation (10), it can be found that if $v_{r}=0$, (10) reduces to $R_{min}^{2}\left(t_{d}\right)=R_{M}^{2}-{\left(R_{0}\tan\theta_{sq}\right)}^{2}=R_{0}^{2}$, which is equal to the static target. On the other hand, for the moving target, $R_{min}\neq R_{0}$, due to the radial velocity $v_{r}.$ According to Equations (4) and (10), the ground range location of the moving target can be expressed as follows:

(13) $$\begin{array}{cc} {\tilde{y}}_{m} & =\sqrt{R_{min}^{2}-H^{2}}\\ & \;=\sqrt{R_{M}^{2}-\left[\frac{v_{r}^{2}R_{M}^{2}}{V^{2}}-\frac{2v_{r}R_{M}R_{0}\tan\theta_{sq}}{V}+{\left(R_{0}\tan\theta_{sq}\right)}^{2}\right]-H^{2}}\;\\ & =\sqrt{y_{m}^{2}-\left(\frac{v_{r}^{2}R_{M}^{2}}{V^{2}}-\frac{2v_{r}R_{M}R_{0}\tan\theta_{sq}}{V}\right)}, \end{array}$$

Consequently, the moving target’s cross-track shift can be expressed as follows:

(14) $$\Delta y_{m}={\tilde{y}}_{m}-y_{m}=-y_{m}+\sqrt{y_{m}^{2}-\left(\frac{v_{r}^{2}R_{M}^{2}}{V^{2}}-\frac{2v_{r}R_{M}R_{0}\tan\theta_{sq}}{V}\right)},$$

Equations (12) and (14) reveal the two-dimensional shift characteristics of a moving target in squint SAR. The along-track shift $\Delta x_{m}$ depends on the target’s radial velocity $v_{r}$ and its slant range $R_{M}$, similar to the side-looking case but with $R_{M}=R_{0}$. Crucially, the cross-track shift $\Delta y_{m}$ is also a function of $v_{r}$, $R_{M}$, the squint angle $\theta_{sq}$, and the target’s true ground range $y_{m}$.

This coupling creates a fundamental challenge: after detecting a moving target, its imaged coordinates $\left(Vt_{d},\;y_{m}\right)$ are known, but its true location $\left(x_{m},y_{m}\right)$ and the associated $R_{M}$ required for relocation via (12) are unknown due to the interdependent shifts in both dimensions. Therefore, the relocation method for side-looking SAR—which primarily corrects the along-track shift based on image-domain parameters—becomes inadequate for squint SAR. A new method capable of resolving this two-dimensional coupling is required, which is developed in the following section.

## 3. A Two-Dimensional Relocation Method for Squint Multichannel SAR

**Figure 2:**
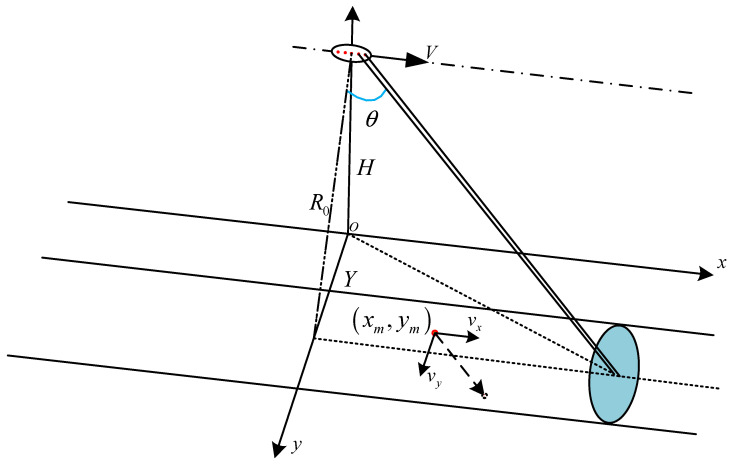
Imaging geometry of the squint multichannel SAR system.

The imaging geometry for the proposed relocation method within a squint multichannel SAR system is shown in Figure 2. The system employs a squint beam configuration with multiple (more than two) receiving channels.

The core of the proposed method lies in resolving the interdependence between the along-track shift $\Delta x_{m}$ and the beam-center slant range $R_{M}$ expressed in Equations (4) and (12). By substituting the expression for the imaged ground range $y_{m}$ from Equation (13) into the definition of $R_{M}$ in Equation (4), we can relate $R_{M}$ directly to the measured parameters and the unknown along-track shift:

(15) $$R_{M}=\sqrt{H^{2}+{\tilde{y}}_{m}^{2}+{\left(R_{0}\tan\theta_{sq}\right)}^{2}+2R_{0}\Delta x_{m}\tan\theta_{sq}+\Delta x_{m}^{2}}.$$

Equations (12) and (15) form a coupled system where the two unknowns, $\Delta x_{m}$ and $R_{M}$, are functions of each other:

(16) $$\left\{\begin{array}{c} {\hat{R}}_{M}=\sqrt{H^{2}+{\tilde{y}}_{m}^{2}+{\left(R_{0}\tan\theta_{sq}\right)}^{2}+2R_{0}\tan\theta_{sq}\;\Delta {\hat{x}}_{m}+\Delta {\hat{x}}_{m}^{2}}\\ \Delta {\hat{x}}_{m}=-\frac{v_{r}}{V}{\hat{R}}_{M} \end{array}\right..$$

Given that the radial velocity $v_{r}$ can be estimated from the multichannel data after clutter suppression, Equation (16) contains only two unknowns. This system of equations can be solved analytically. Substituting the second equation into the first yields a quadratic equation in $R_{M}$. Solving and retaining the physically meaningful positive root leads to the following explicit expressions for the estimated along-track shift $\Delta {\hat{x}}_{m}$ and the estimated beam-center slant range ${\hat{R}}_{M}$:

(17) $$\left\{\begin{array}{c} \Delta {\hat{x}}_{m}=-\frac{v_{r}\left[\sqrt{{\left(v_{r}R_{0}\tan\theta_{sq}\right)}^{2}+\left(V^{2}-v_{r}^{2}\right)\left[H^{2}+{\tilde{y}}_{m}^{2}+{\left(R_{0}\tan\theta_{sq}\right)}^{2}\right]}-v_{r}R_{0}\tan\theta_{sq}\right]}{V^{2}-v_{r}^{2}}\\ {\hat{R}}_{m}=\frac{V\left[\sqrt{{\left(v_{r}R_{0}\tan\theta_{sq}\right)}^{2}+\left(V^{2}-v_{r}^{2}\right)\left[H^{2}+{\tilde{y}}_{m}^{2}+{\left(R_{0}\tan\theta_{sq}\right)}^{2}\right]}-v_{r}R_{0}\tan\theta_{sq}\right]}{V^{2}-v_{r}^{2}} \end{array}\right..$$

Equation (17) shows that the two-dimensional location shift of the moving target depends solely on its estimated radial velocity $v_{r}$ and its imaged cross-track location $y_{m}$, in addition to the known system parameters $\left(V,\;{H,\;R}_{0},\;y_{m},\;\theta_{sq}\right)$. The true cross-track location $y_{m}$ is implicitly accounted for through its relationship with $y_{m}$ and $v_{r}$ in Equation (13). For the side-looking case $\theta_{sq}=0$, Equation (17) simplifies to the known one-dimensional relocation solution [4,10].

Once $\Delta {\hat{x}}_{m}$ and ${\hat{R}}_{m}$ are obtained from Equation (17), the true geographic coordinates of the moving target can be recovered. The true along-track location ${\hat{x}}_{m}$ is found by correcting the imaged azimuth position $x_{m}$ with the estimated shift. The true cross-track location ${\hat{y}}_{m}$ is derived from the geometry defined in Equation (4):

(18) $$\left\{\begin{array}{c} {\hat{x}}_{m}={\tilde{x}}_{m}+\Delta {\hat{x}}_{m}\\ {\hat{y}}_{m}=\sqrt{{\hat{R}}_{m}^{2}-H^{2}-{\left(R_{0}\tan\theta_{sq}\right)}^{2}} \end{array}\right..$$

**Figure 3:**
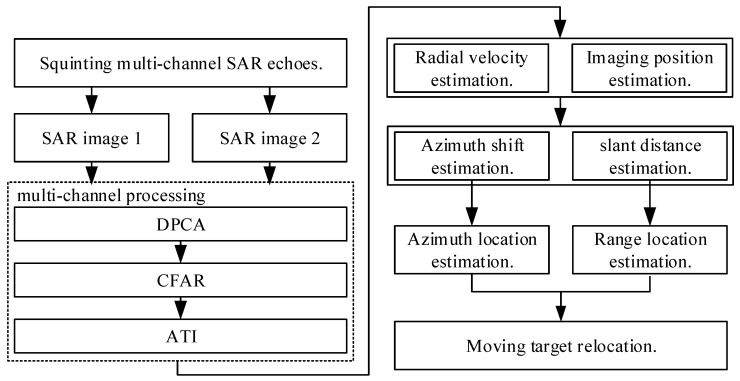
Flowchart of the proposed moving-target relocation algorithm for squint multichannel SAR.

Based on the above derivation, a complete workflow for moving target relocation in squint multichannel SAR is proposed, as outlined in the flowchart in Figure 3. The procedure consists of the following steps:

Step 1 (SAR Imaging): The raw echo data received by each channel are first range-compressed and then azimuth-focused using a standard squint SAR imaging algorithm (e.g., a squint-mode Range–Doppler or Chirp Scaling processor). During this process, the range–azimuth coupling caused by the squint angle is properly handled, ensuring that stationary scene targets are well-focused. The output is a set of complex-valued single-look SAR images, one per receiving channel, aligned to a common image grid.

Step 2 (Clutter Suppression & Detection): In the image domain, a multichannel clutter suppression technique, such as displaced phase center array (DPCA) or space-time adaptive processing (STAP), is applied to complex images. This operation suppresses stationary clutter while preserving moving target signals. After clutter suppression, a constant false alarm rate (CFAR) detector [18] scans the residual image to identify pixels that likely contain moving targets, yielding a set of detected target positions.

Step 3 (Parameter Estimation): For each detected target, the radial velocity $v_{r}$ is estimated from the phase differences among the clutter-suppressed multichannel signals. Typical methods include along-track interferometry (ATI) or phase-comparison monopulse, which convert the interferometric phase into a radial velocity estimate using the known platform velocity and baseline geometry. Simultaneously, the imaged coordinates $\left({\tilde{x}}_{m},{\tilde{y}}_{m}\right)$ of the target are recorded from the detection output.

Step 4 (Shift & Slant Range Calculation): Calculate the targets’ along-track shift $\Delta {\hat{x}}_{m}$ and the beam-center slant range ${\hat{R}}_{M}$ using Equation (17), with inputs $v_{r}$, $y_{m}$, and the system parameters. These expressions directly resolve the coupled relationship between the along-track shift and the slant range at the beam-center moment.

Step 5 (True Location Estimation): Compute the target’s true along-track and cross-track locations $\left({\hat{x}}_{m}{,\hat{y}}_{m}\right)$ using Equation (18).

Step 6 (Relocation & Output): Finally, the estimated true locations $\left({\hat{x}}_{m}{,\hat{y}}_{m}\right)$ of all detected moving targets are marked on the SAR image or output as a geolocation list, completing the two-dimensional relocation of moving targets in the squint SAR system.

## 4. Simulation and Analysis

### 4.1. Effects of System Parameters for Ground Moving Target Relocation

This section quantitatively analyzes the impact of key system and target parameters on the cross-track shift derived in Equation (14). The simulation parameters for the squint SAR system are listed in Table 2. Equation (14) indicates that the cross-track shift $\Delta y_{m}$ is a function of the target’s true cross-track location $y_{m}$, radial velocity $v_{r}$, platform velocity V, and the squint angle θ.

**Figure 4:**
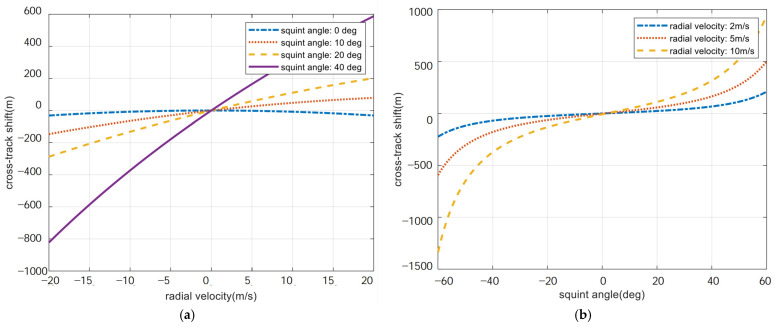
Effect of system and target parameters on cross-track shift. (**a**) Cross-track shift versus radial velocity for different squint angles. (**b**) Cross-track shift versus squint angle for different radial velocities.

Figure 4 illustrates the effects of the squint angle and radial velocity on the cross-track shift for a target at a fixed ground range. As shown in Figure 4a, when the squint angle is zero (side-looking mode), the cross-track shift remains zero regardless of the radial velocity. However, for any non-zero squint angle, the cross-track shift increases significantly with increasing radial velocity. Conversely, Figure 4b demonstrates that for a given radial velocity, the cross-track shift grows substantially as the squint angle increases.

**Figure 5:**
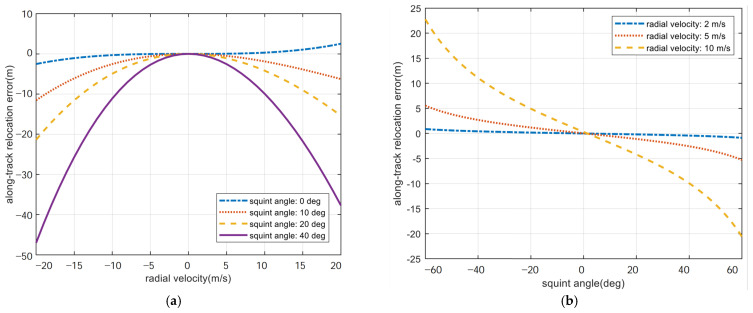
Along-track relocation error induced by neglecting the cross-track shift. (**a**) Along-track relocation error changes with the radial velocity. (**b**) Along-track relocation error changes with the squint angle.

Furthermore, applying the along-track relocation method to a ground moving target in a squint SAR system without compensating for its cross-track shift introduces substantial errors even in the along-track direction. As shown in Figure 5, the along-track location error exhibits a positive correlation with the change in the squint angle and radial velocity of the target. When the moving target’s radial velocity is 20 m/s, the along-track relocation error can reach 20 m, 48 m, and 97 m in the squint SAR system with squint angles of 20°, 40°, and 60°, respectively. Therefore, the cross-track shift is marginal only at low squint angles. At high squint angles, the effect of cross-track shift must be considered.

Furthermore, applying a traditional along-track-only relocation method based solely on Equation (12) in a squint SAR system, without compensating for the cross-track shift, introduces significant errors even in the along-track direction. This is because the uncorrected cross-track shift leads to an erroneous estimation of $R_{M}$ in Equation (12). Figure 5 quantifies this along-track relocation error. The error exhibits a strong positive correlation with both the target’s radial velocity and the system’s squint angle. For instance, when the radial velocity is 20 m/s, the along-track error can reach approximately 20 m, 48 m, and 97 m for squint angles of 20°, 40°, and 60°, respectively. These results underscore that the cross-track shift is negligible only at very low squint angles. For moderate to high squint angles, a full two-dimensional relocation is essential.

### 4.2. Validation of the Proposed Relocation Algorithm

To validate the efficacy and accuracy of the proposed relocation method, this section presents the relocation results for moving targets in a squint SAR system. The system parameters are those listed in Table 1. The imaged cross-track location ${\tilde{y}}_{m}$ was calculated based on the coordinate system illustrated in Figure 1. The initial parameters of the moving targets are presented in Table 3, and the results of the radial velocity estimation for these targets are detailed in Table 4.

**Figure 6:**
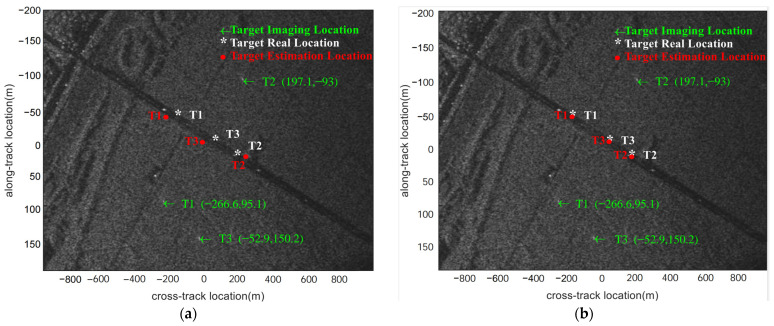
Moving target relocation results obtained using two different methods. (**a**) Relocation results obtained using traditional methods that ignore the cross-track shift. (**b**) Relocation results obtained using the proposed methods that consider the cross-track shift.

In the experiment, the displaced phase center antenna (DPCA) and along-track interferometry (ATI) methods were combined to detect moving targets and estimate their radial velocities. The imaged locations of the moving targets are marked as blue arrows in Figure 6, showing noticeable deviations from their true locations, while the final relocation results obtained by different methods are represented by red dots.

Two relocation methods were compared. The traditional algorithm [11] ignores the two-dimensional range–azimuth shift caused by squint, resulting in significant relocation errors even with a highly accurate radial velocity estimate. Under this one-dimensional approach, the true cross-track location is assumed to be directly given by the imaged location, and the along-track shift is then calculated using Equation (12). Figure 6a shows the results, where the relocated positions still diverge significantly from the true locations.

In contrast, the approach presented in this paper accounts for the coupled shift in both along-track and cross-track directions. Substituting the imaged two-dimensional position and the estimated radial velocity into Equation (17) yields the true location. The relocation result is shown in Figure 6b, where the marker aligns precisely with the center of the road, confirming accurate geolocation.

To further quantify the performance, a single-scene relocation experiment with three targets was conducted. Using the traditional method that ignores the cross-track shift, the relocated coordinates for targets T1, T2, and T3 were (−266.57 m, 38.02 m), (197.14 m, 22.89 m), and (−52.92 m, 1.01 m), respectively. In comparison, the proposed method produced coordinates that accurately matched the true locations. The primary source of residual estimation error is the measurement error in the target’s imaged location. The relocation error results are presented in Table 5. As shown, the relocation errors of the proposed algorithm are substantially smaller than those of the conventional algorithm, demonstrating a clear performance advantage, particularly in range relocation. Consistent with the previous analysis, it is evident that the conventional algorithm produces relatively small azimuth positioning errors, whereas its range positioning error increases progressively with the squint angle. These simulation results demonstrate the effectiveness and accuracy of the proposed method. Therefore, the traditional along-track relocation algorithm may still be applicable to squint SAR systems with very small squint angles and predominantly slow-moving targets. However, for general cases involving significant squint angles, the proposed two-dimensional method is necessary to achieve precise relocation.

**Figure 7:**
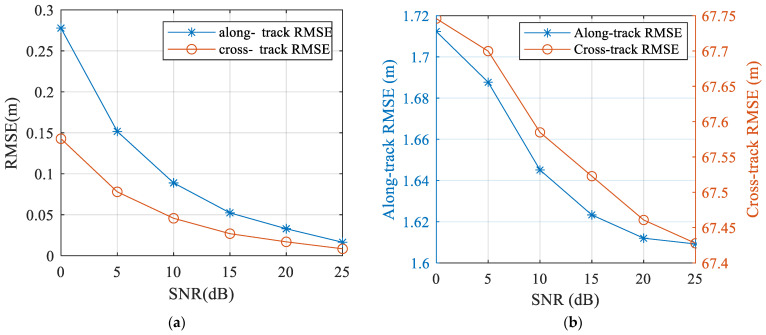
Relocation RMSE of the proposed algorithm and traditional method under different SNRs. (**a**) Proposed method. (**b**) Traditional method.

To further assess the robustness of the proposed algorithm, the following experiment evaluated the root mean square error (RMSE) of both the proposed and the conventional algorithms under different signal-to-noise ratios (SNRs). Here, SNR refers to the signal-to-noise ratio before clutter suppression. For fair comparison, the RMSE of each method was computed over 100 Monte Carlo trials and is shown in Figure 7.

Because the conventional method produces substantially larger errors in the range direction, a dual-axis plot was used in Figure 7b to present the results clearly: the left vertical axis represents the along-track RMSE, and the right vertical axis represents the cross-track RMSE. It can be observed that the conventional algorithm exhibits not only a larger along-track RMSE than the proposed method, but also a drastically larger cross-track RMSE across the entire range of SNRs. As the SNR decreases, the relocation accuracy of both methods degrades somewhat, yet the proposed algorithm maintains relatively low errors in both directions. Conversely, as the SNR increases, the RMSE of the proposed algorithm gradually decreases. These results confirm the effectiveness and robustness of the proposed algorithm under varying SNR conditions, and further highlight the necessity of accounting for two-dimensional displacements for accurate relocation.

## 5. Conclusions

This paper presented a novel relocation algorithm for ground moving targets within a squint multichannel SAR framework. The proposed method effectively addresses the two-dimensional shift in squint SAR imagery, where moving targets exhibit coupled along-track and cross-track shifts. By establishing an analytical model that links the imaged position to the true location and radial velocity, the algorithm enables precise geolocation by solving the coupled equations.

The experimental results demonstrate that the along-track positioning accuracy of the proposed algorithm is comparable to that of the conventional algorithm, whereas the range positioning accuracy is significantly higher, with the relative error reduced substantially. Moreover, this accuracy advantage becomes increasingly pronounced as the squint angle increases, further confirming the necessity of two-dimensional relocation in squint SAR systems.

Future work will first aim to validate the proposed algorithm with real multi-channel squint SAR data as they become available. In addition, extending the method to multi-frame joint relocation and tracking, potentially through sequential estimation frameworks such as the extended Kalman filter, represents another important direction.

## Figures and Tables

**Table 1:** Nomenclature.

Symbol	Description
*θ_sq_*	Radar beam squint angle
*V*	Platform velocity
*H*	Flight altitude
*Y*	Shortest ground range from radar to scene center
$v_{x}$	Along-track velocity component of the moving target
$v_{y}$	Cross-track velocity component of the moving target
$R_{0}$	Shortest range from the radar to the scene center
*t*	Slow time
$R_{M}$	Slant range from radar to the moving target at *t* = 0
$\left(x_{m},y_{m}\right)$	True ground coordinates of the moving target
$\left({\tilde{x}}_{m},{\tilde{y}}_{m}\right)$	Imaged coordinates of the moving target
$\left({\hat{x}}_{m}{,\hat{y}}_{m}\right)$	Estimated true coordinates of the moving target
$\left(\Delta x_{m},\Delta y_{m}\right)$	Moving target’s along- and cross-track shifts in the SAR image

**Table 2:** SAR radar system parameters.

System Parameter	Value
Wavelength	0.03 m
Pulse repetition frequency	1024 Hz
Carrier velocity	200 m/s
Shortest slant range of scene center	5000 m
Shortest ground range of scene center	4000 m
Flight altitude	3000 m

**Table 3:** Initial parameters of moving targets.

Target	Along-Track Location (m)	Cross-Track Location (m)	Along-Track Velocity (m/s)	Cross-Track Velocity (m/s)
T1	−39.30	−199.20	−1.20	−6.50
T2	22.10	148.50	0.80	5.20
T3	−0.50	18.80	−1.10	−7.00

**Table 4:** Radial velocity estimation results of moving targets.

Target	Radial Velocity (m/s)
T1	−5.1981
T2	4.2382
T3	−5.6482

**Table 5:** Location errors of the traditional and proposed method.

Target	Traditional Method		Proposed Method	
	Along-Track Error (m)	Cross-Track Error (m)	Along-Track Error (m)	Cross-Track Error (m)
T1	1.37	−69.73	0.28	−0.36
T2	0.92	49.75	0.22	−0.23
T3	1.77	−72.12	0.27	−0.43

## Data Availability

The data presented in this study are available on request from the corresponding author due to legal restrictions.
